# Analgesic Effects of Oxycodone in Combination With Risperidone or Ziprasidone: Results From a Pilot Randomized Controlled Trial in Healthy Volunteers

**DOI:** 10.3389/fpain.2022.752256

**Published:** 2022-02-04

**Authors:** Ameet S. Nagpal, Daniel J. Lodge, Jennifer S. Potter, Alan Frazer, Robin Tragus, Megan E. Curtis, Angela M. Boley, Maxim Eckmann

**Affiliations:** ^1^Department of Anesthesiology, UT Health San Antonio, San Antonio, TX, United States; ^2^Department of Pharmacology, UT Health San Antonio, San Antonio, TX, United States; ^3^Department of Psychiatry, UT Health San Antonio, San Antonio, TX, United States; ^4^Department of Pediatrics, UT Health San Antonio, San Antonio, TX, United States; ^5^Department of Psychology, University of Texas at San Antonio, San Antonio, TX, United States

**Keywords:** opioid, oxycodone, antipsychotic, abuse, risperidone, ziprasidone

## Abstract

**Background and Objectives:**

Patients taking opioids are at risk of developing dependence and possibly abuse. Given the role of the mesolimbic dopamine system in opioid reward, blocking dopamine D2 receptors should limit the abuse liability of opioid analgesics. This pilot study evaluates the analgesic efficacy of oxycodone combined with an atypical antipsychotic (dopamine D2 receptor antagonist).

**Methods:**

A randomized, double-blind, within-subjects, controlled trial in healthy volunteers was conducted at UT Health SA Pain Clinic. Fifteen volunteers with previous medical exposure to opioids were enrolled. Risperidone (2 mg) or ziprasidone (80 mg) in combination with oxycodone (5, 10, 15 mg) was administered. Pain intensity using the cold pressor test, Current Opioid Misuse Measure (COMM), Addiction Research Center Inventory (ARCI, opioid subscale), Drug likability with drug effects questionnaire (DEQ) were assessed.

**Results:**

Oxycodone produced dose dependent increases in thermal analgesia on the cold pressor test that was significant at 10 and 15 mg (*t* = 3.087, *P* = 0.017). The combination did not significantly alter thermal analgesia. There was no significant effect of the combination on the ARCI or the POMS.

**Discussion and Conclusion:**

The combination of an atypical antipsychotic with oxycodone does not alter analgesic response or increase the incidence of adverse effects when compared to oxycodone alone. Such information is critical for the development of drug combinations for the treatment of pain and provide the foundation for future studies of abuse potential in drug users.

**Scientific Significance:**

This intervention in chronic pain patients is unique because it utilizes FDA approved drugs in combination to reduce abuse liability. The first step, and aim of this study, is to confirm the drug combination does not interfere with analgesic efficacy. The next step is to examine the combination in recreational drug users to assess the potential to block the euphoric effects of oxycodone. Ultimately, if this combination is effective, this approach could be beneficial in management of chronic pain.

## Background and Objectives

Opioids remain one of the most abused substances in the United States of America (USA) (1). Among patients who receive long-term opioid prescriptions, one in four will become dependent which could possibly lead to substance use disorder (2–7). Such abuse potential, coupled with a risk of respiratory depression, has contributed to the opioid epidemic. The US Department of Health and Human Services (HHS) five-point Strategy to Combat the Opioid Crisis calls for better pain treatments (8). Therefore, we have examined a novel method to reduce the abuse potential of prescription opioids without altering their analgesic efficacy by administering a fixed-dose-combination of an opioid with an atypical antipsychotic drug. This initial study was carried out with healthy volunteers to focus on the safety of the drug combination as well as evaluating if the combination produced comparable analgesia as an opioid alone.

Research suggests the rewarding properties of opioids are mediated, in part, by their ability to increase dopamine in brain regions such as the nucleus accumbens (9–15). Preclinical studies demonstrate that by blocking dopaminergic D2 receptors, antipsychotic agents reduce the rewarding properties of commonly abused drugs (16–21), including opioids (22–24). Unfortunately, in clinical settings, antipsychotics have generally been found to be ineffective in maintaining abstinence from psychostimulant use (25). There are a number of reasons for this, including a lack of patient compliance. Indeed, individuals with substance use disorders are likely to discontinue medications that decrease the euphoric effects of a drug if their original intention is intoxication (26). Thus, a fixed-dose combination would be beneficial to maintain patient compliance. It should be noted that the maintenance of drug-taking behavior may be regulated by psychosocial factors, such as craving and impulsivity, involving regions outside of the dopamine system (12, 15, 27, 28).

Atypical antipsychotics are less likely to produce the severe extrapyramidal symptoms than typical antipsychotics (29–32), making them a better choice, although they may have metabolic adverse effects that may be a potential compliance-impacting problem for long term use (33). For the current study, we examined two distinct antipsychotic drugs, risperidone and ziprasidone. As with most atypical antipsychotic drugs, these compounds possess a promiscuous pharmacology; however, risperidone is an antagonist at the dopamine D1 and D2 receptors whereas ziprasidone is an antagonist at the D2 receptor. They are commonly prescribed drugs in the USA, with a significant amount of clinical data to evaluate potential drug-induced contraindications. Whereas, a true fixed-dose-combination would require Federal Drug Administration (FDA) approval as an Investigational New Drug (IND) in the USA, the concurrent administration of these two drugs does not and permits the examination of the subjective and analgesic response of this combination in healthy volunteers.

The primary goal of this double-blind, placebo controlled, randomized, crossover study was to examine the analgesic efficacy of an opioid (oxycodone) administered concurrently with an atypical antipsychotic (risperidone or ziprasidone) compared to those produced by the opioid alone (plus placebo) in healthy volunteers. Doses were determined based on the clinical analgesic potency of oxycodone and D2 receptor occupancy of the antipsychotic (34–37). The hypothesis is the antipsychotic and opioid drug combination will not significantly reduce the analgesic efficacy of oxycodone in healthy volunteers. Subsequent studies will evaluate the abuse potential of this combination in recreational drug users.

## Methods

The research study was approved by the University of Texas Health Science Center at San Antonio's Institutional Review Board (IRB). All participants signed an informed consent and were compensated for their time through a reloadable debit card.

### Participants

Healthy volunteers (*n* = 15), 8 (53.3%) female and 7 (46.7%) male, who had previous medical exposure to opioids (de-risking the potential for abuse following initial opioid exposure) were recruited. Participants were recruited by flyers placed at local cafes, universities, and on the University of Texas Health Science Center at San Antonio research volunteer recruiting system (http://vpr.uthscsa.edu/findastudy/). Participants were enrolled by the research team and assigned to interventions based on the randomization schedule.

Individuals were excluded if they reported at least one of the following: a psychiatric comorbidity; a chronic pain disorder; a history of substance use disorder; a history or presence of diabetes or cardiac disease or arrhythmia; current analgesic or neuroleptic medication; a positive pregnancy or drug urine test; or score of ≥9 on the Current Opioid Misuse Measure (38).

### Study Design

This pilot study was a double-blind, randomized, within-subjects, controlled trial design, conducted between June 2017 and June 2018 (NCT04587115, October 2020, retrospectively registered, https://clinicaltrials.gov/ct2/show/NCT04587115) with no important changes to the methods after trial commencement. A sample size of 15 subjects (5/group) was sought without sample size justification. The study was stopped when sufficient participants were enrolled and completed. The study consisted of three visits to the University of Texas Health San Antonio Pain Clinic. Visits 2 and 3 were at least 48 h apart to allow for medication washout and within-subjects comparisons. A urine sample was collected at the beginning of each visit to determine the presence of illicit drugs and pregnancy (females only). After the baseline visit, participants were randomized with a 1:1 allocation to receive first either a dose of oxycodone plus placebo or a dose of oxycodone plus either of the two antipsychotics, risperidone (2 mg) or ziprasidone (80 mg). Participants were randomized to receive either risperidone or ziprasidone in a 1:1 ratio. The randomization schedule was generated by the research team and kept at the pharmacy, and the drug was delivered in a bottle labeled “Oxycodone Clinical Trial” and by participant name and visit number to keep the researchers and volunteers blinded. To determine safety and efficacy, three different doses of oxycodone were examined in separate groups of participants (i.e., a participant received either 5, 10, or 15 mg of oxycodone).

### Baseline (Visit 1)

After participants completed assessments, they became familiar with validated experimental pain procedures (cold pressor task and thumb pressor task) (39, 40) and established baseline pain thresholds. After completing the experimental pain procedures, participants were debriefed and scheduled for visits 2 and 3. After participants completed visit 1, a study physician wrote two triplicate prescriptions that read “Oxycodone Clinical Trial” and were dropped off at a local compounding pharmacy. The pharmacy was unblinded to the study and maintained the randomization schedule.

### Visits 2 and 3

Study visits 2 and 3 lasted 3-4 h. Participants orally consumed the study medication in front of the research assistant. Participants' subjective ratings of the study medication, pain threshold, and pain tolerance/severity were assessed 60 and 120 min after medication consumption. The Abnormal Involuntary Movement Scale (AIMS) (41) and the Barnes Akathisia Rating Scale (BARS) (42) were administered by the attending physician to determine discharge readiness. Once the patient was ready for discharge, the patient was debriefed from the study. If adverse events were experienced, study physicians were notified immediately, and the IRB was notified.

### Experimentally Induced Pain

The experimental pain procedures were completed during baseline visit, and during visits 2 and 3 at 60 and 120 min after medication consumption.

### Cold Pressor Task

Participants completed a cold pressor task by submerging their non-dominant hand up to their wrist in a circulating water bath (ARCTIC Series Refrigerated Bath Circulators Haake SC 100) at 4.0 ± 0.1°C for up to 300 s. Participants sat comfortably, facing away from the device and experimenter to minimize any distractions. Pain severity was verbally collected every 30 s through a visual analog scale (0 = No pain to 100 = Worst pain imaginable). Pain threshold was defined as the duration the non-dominant hand remain submerged in the water bath (39, 43).

### Thumb Pressure Task

In addition to the cold pressor task, participants completed a pressure stimulation, whereby a pressure algometer was applied to the dominant hand's thumbnail to determine pain tolerance and threshold in response to mechanical stimulation. Pressure was applied for 5 s intervals followed by a 20 s release of pressure. Weight started at an initial stimulus of 0.5 kg/cm^2^ and was increased by 0.5 kg/cm^2^ increments, up to a maximum of 10 kg/cm^2^ (19). Participants were instructed to rate their pain severity (VAS, 0-100) after each time the pressor was released from their thumb (43). The procedure was stopped once the participant verbally said “stop” and indicated their pain was intolerable.

### Study Medication Combination

The study medication combination was compounded by Oakdell Pharmacy, a licensed pharmacy with a history of being involved in research protocols in the South Texas region. Study medication was compounded into a single maroon opaque size #0 capsule (with microcrystalline cellulose used to fill the space in the capsule) according to the randomization schedule. The pharmacy delivered the study medication to the participant at the beginning of each study visit.

Risperidone (2 mg) and ziprasidone (80 mg) were selected as the atypical antipsychotic drugs to be used in combination with the opioid. Both are antagonists at the dopamine D2 receptor. Compared to risperidone, ziprasidone has a better side-effect profile and is metabolized primarily by CYP3A4. However, the Tmax for ziprasidone (~4 h) (44) is longer than that of oxycodone IR (<2 h), so its ability to block the euphoric effect of oxycodone could be delayed, especially for single dose studies. Consequently, for the current study we used a moderately high dose of ziprasidone, to ensure appreciable D2 receptor blockade when peak plasma concentrations of oxycodone occur. By contrast, the Tmax of risperidone (<2 h) more closely matches oxycodone (<2 h), although its side effect profile and variability in CYP2D6 metabolism between individuals is not ideal. Variations in CYP2D6 contribute to an increased risk of adverse events such as nausea, QTc prolongation, increased plasma levels, or poor efficacy (45, 46); while pharmacokinetic effects were not measured for this study they are planned for future studies.

## Measures

Study data were collected and managed using REDCap electronic data capture tools hosted at UT Health San Antonio (47, 48). REDCap (Research Electronic Data Capture) is a secure, web-based software platform designed to support data capture for research studies, providing (1) an intuitive interface for validated data capture; (2) audit trails for tracking data manipulation and export procedures; (3) automated export procedures for seamless data downloads to common statistical packages; and (4) procedures for data integration and interoperability with external sources.

During visit 1, a demographics form was administered to obtain sex, date of birth, ethnicity, and education. A history of substance use disorder was determined by substance use within the past 30 days and years of lifetime use (a checklist of different drugs such as alcohol, cigarettes, pain medications, and illicit drugs, including a checkbox for route of administration of drug). A current health status self-report of medical history and medication history was provided to determine eligibility of study participation. Questions related to chronic pain were administered to determine pain severity and inference of daily functioning. The Current Opioid Misuse Measure (COMM) is a common measure and was used to determine whether a participant was exhibiting aberrant behaviors associated with misuse of opioid medications (49).

The following surveys were all administered at baseline and during visits 2 and 3 at 60 and 120 min after initial study drug consumption.

### Pain Intensity

A Visual Analog Scale (VAS) was used to measure pain intensity. Participants were instructed to rate their pain from 0 “no pain” to 100 “worst pain imaginable” (50).

### Addiction Research Center Inventory

The ARCI (opioid subscale) is a 49-item self-administered assessment. The ARCI measures euphoria and other drug effects at the time of exposure and asks participants to respond “true” or “false” to a series of statements about their experiences after drug administration (51).

### Drug Likability

The Drug Effects Questionnaire is a five-item self-administered assessment where participants place a mark on a line from 0 to 100, with 0 being none and 100 being extremely for each question (29).

### Mood State

The Profile of Mood States (POMS) is a 65-item mood adjective checklist in which each adjective is scored from 0 “absent” to 4 “very much” (52).

### Physician Assessment Readiness for Discharge

To determine whether participant was ready to be discharged, study physicians completed the Abnormal Involuntary Movement Scale (AIMS) (41) and Barnes Akathisia Rating Scale (BARS) (42). Participants with an Aldrete score of 10/10 were considered safe for discharge.

## Data Analysis

The analgesic effect of oxycodone alone or in combination with an antipsychotic was analyzed by a Two-Way Repeated Measures (RM) Analysis of Variance (ANOVA) with dose of oxycodone and drug as factors. The effects of these drugs on the ARCI and POMS were analyzed by a Three-Way ANOVA with dose of oxycodone, drug, and subscale as factors. To examine the dose-dependent effects of oxycodone on VAS measures of drug liking, a one-way RM ANOVA comparing, baseline, with three doses of oxycodone was used.

Given that this was a preliminary study with limited information about the magnitude of the purported effect, we were unable to conduct a power analysis to determine group sizes. Indeed, a secondary goal of this study was to obtain such information to guide future clinical experiments.

## Results

Participants identified as White/European (*n* = 10; 66.7%), Spanish/Hispanic/Latino (*n* = 4, 26.7), or Asian or Pacific Islander (*n* = 1, 6.7%). Most participants' highest level of education was bachelor's degree (*n* = 13, 86.7%), followed by some time in college, technical school or Associate's Degree (*n* = 1, 6.7%), and graduate degree (*n* = 1, 6.7%).

**Figure 1:**
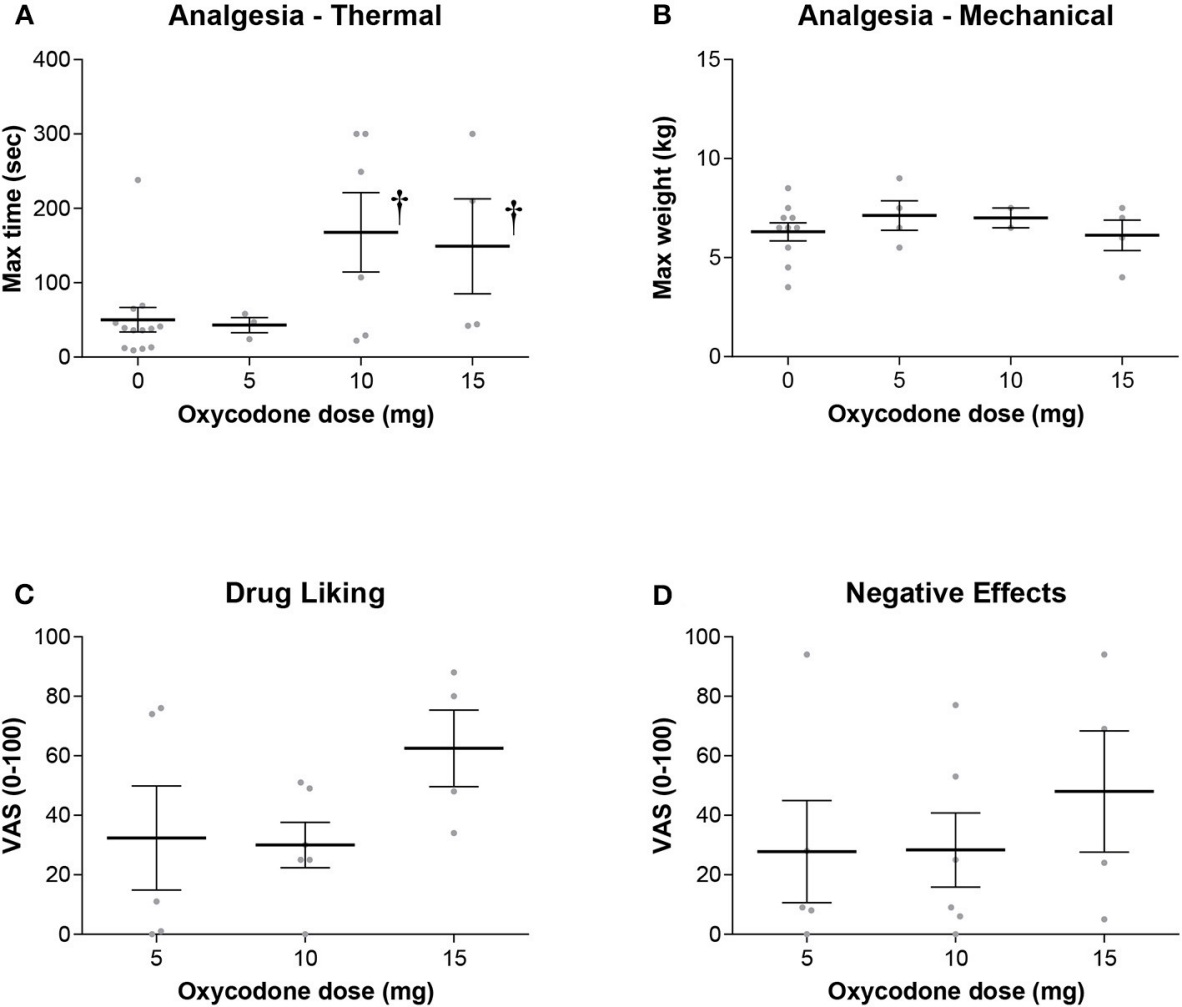
**(A)** Oxycodone alone produced a dose dependent increase in thermal analgesia determined by cold-water tolerance. Analgesia was significantly increased at the 10 and 15 mg dose as measured by the time hand was submerged in the water bath (†- *t* = 3.087, *P* = 0.017). There were no significant effects on either **(B)** mechanical analgesia, **(C)** subjective ratings of drug liking, or **(D)** negative effects.

**Figure 2:**
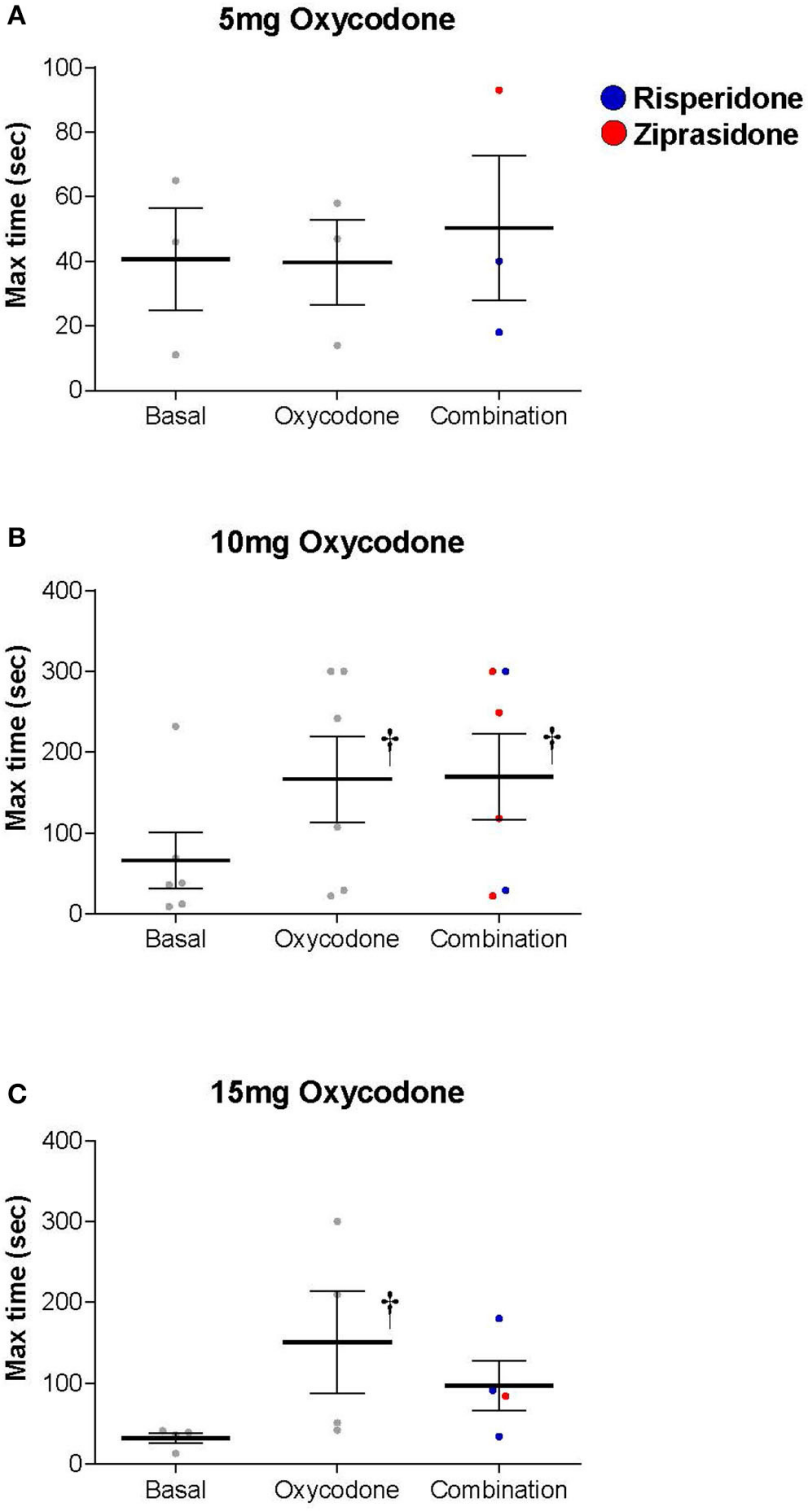
Both Oxycodone/Placebo (Oxycodone) and Oxycodone/Antipsychotic [Combination, (Risperidone-blue, Ziprasidone-red)] produced a dose dependent increase in thermal analgesia determined by cold-water tolerance and the drug combination did not dramatically alter the thermal analgesic response when compared to oxycodone alone (Two Way Repeated Measures ANOVA: Oxycodone/Placebo vs. Oxycodone/Antipsychotic *t* = 0.656, *P* = 0.519). Oxycodone produced an analgesic response at both **(B)** 10 mg (*t* = 3.003, *P* = 0.014) and **(C)** 15 mg (*t* = 2.839, *P* = 0.03) as measured by the time hand was submerged in water bath, and that was not statistically different to that obtained with the drug combination at any dose (5 mg: *t* = 0.0612, *P* = 0.995, 10 mg: *t* = 0.0199, *P* = 0.984, 15 mg: *t* = 1.258, *P* = 0.223). † denotes a significant change from basal level.

Oxycodone produced a dose-dependent increase in maximum thermal analgesia (the maximum thermal analgesia in seconds was recorded at either 60 min or 120 min after drug administration—whichever was highest, Figure 1). This effect was statistically significant at doses of 10 and 15 mg with a 2.5-3.0-fold increase in the latency to remove the hand from the cold water (Two-Way RM ANOVA: Oxycodone/Placebo vs. Baseline *t* = 3.087, *P* = 0.017). Two subjects (both in the 5 mg group) had baseline thresholds exceeding the maximum permitted time (300 s) and were excluded from the analysis. As oxycodone alone did not produce mechanical analgesia, we did not show the data for the effect of oxycodone with an antipsychotic on this measure. Indeed, oxycodone alone was without significant effects on either maximum mechanical analgesia (One Way RM ANOVA: *F* = 1.167, *P* = 0.388), subjective ratings of drug liking (One Way ANOVA: *F* = 1.771, *P* = 0.212) or maximum negative effects (One Way ANOVA: *F* = 0.449, *P* = 0.649). Importantly, and consistent with our overarching hypothesis, the combination of an opioid with an antipsychotic did not dramatically alter the maximum thermal analgesic response when compared to oxycodone alone (Figure 2, Two Way RM ANOVA: Oxycodone/Placebo vs. Oxycodone/Antipsychotic *t* = 0.656, *P* = 0.519). Please note that, for clarity, data with oxycodone alone is included in both Figures 1, 2. Specifically, in the cold water pressor test, oxycodone produced an analgesic response at both 10 mg (*t* = 3.003, *P* = 0.014, Figure 2) and 15 mg (*t* = 2.839, *P* = 0.03) that was not significantly different from that obtained with the drug combination at any dose (5 mg: *t* = 0.0612, *P* = 0.995, 10 mg: *t* = 0.0199, *P* = 0.984, 15 mg: *t* = 1.258, *P* = 0.223).

**Figure 3:**
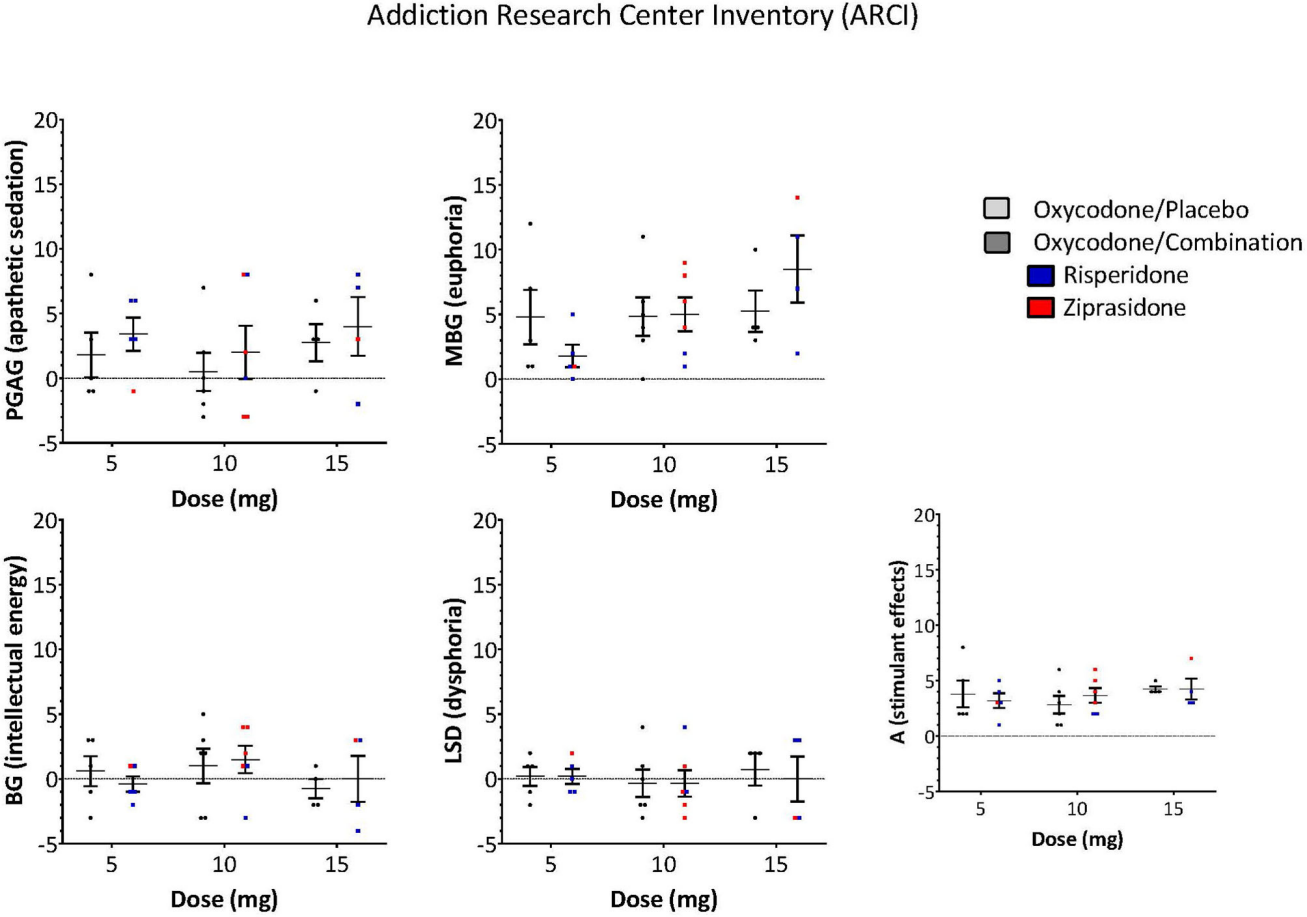
ARCI responses of opioid subscale measurements of drug effects did not differ between oxycodone/placebo and oxycodone/combination (Drug: *F* = 0.243, *P* = 0.623, Drug × Subscale: *F* = 0.374, *P* = 0.827, Drug × Dose × Subscale: *F* = 0.560, *P* = 0.809). [PCAG-index of apathetic sedation, MBG-index of euphoria, BG-index of energy, LSD-index of dysphoria, A-index of alcohol specific effects, (Risperidone-blue, Ziprasidone-red)].

**Figure 4:**
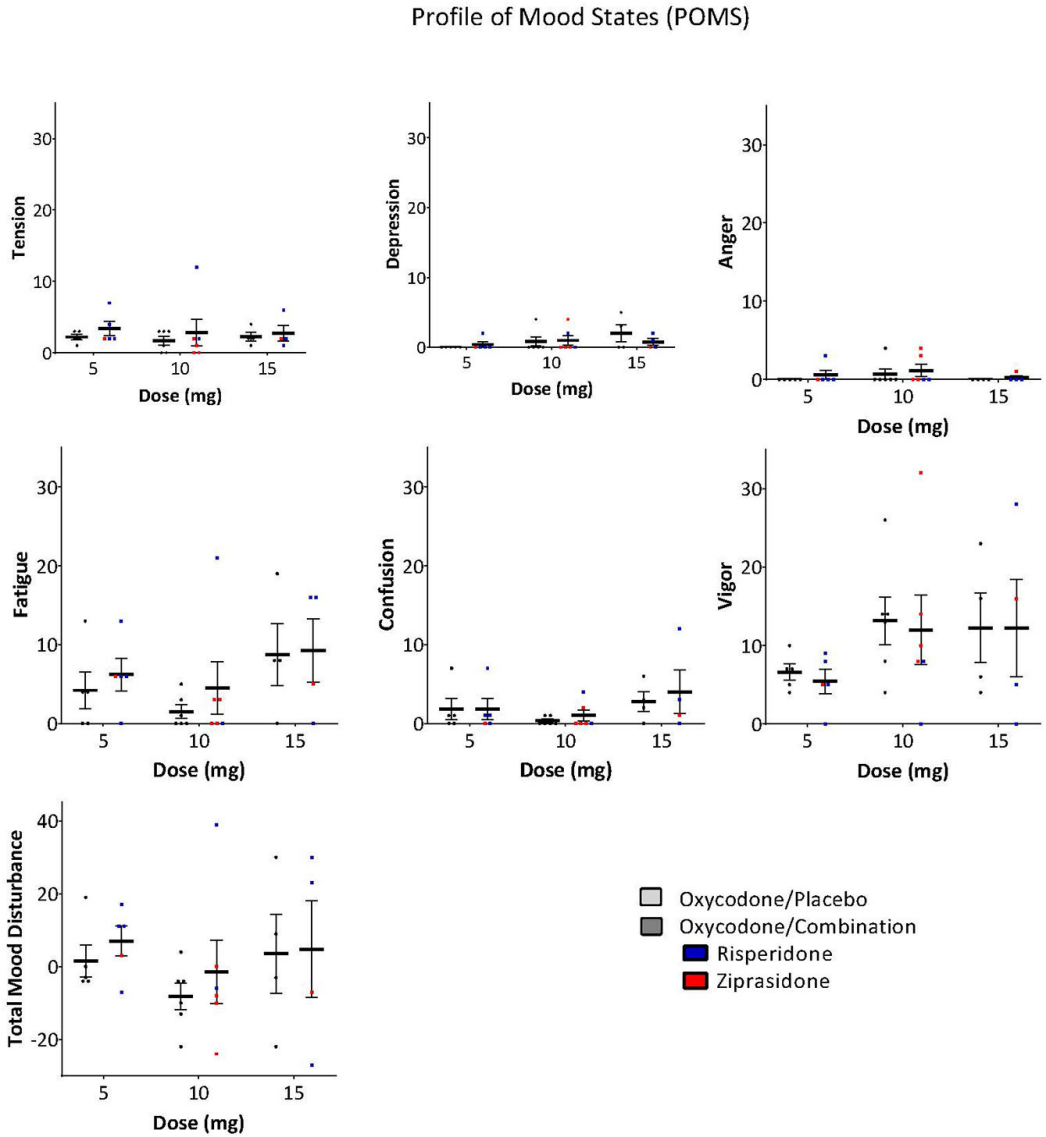
POMS scores on subscales and total mood disturbance were not significantly different between oxycodone/placebo and oxycodone/combination [Drug: *F* = 0.976, *P* = 0.325, Drug × Subscale: *F* = 0.250, *P* = 0.959, Drug × Dose × Subscale: *F* = 0.0361, *P* = 1.000, (Risperidone-blue, Ziprasidone, red)].

The ARCI and POMS were used to examine whether the antipsychotic altered the subjective effects of oxycodone. A Three-Way ANOVA with drug, dose and subscale as factors demonstrated that there was no significant effect of the drug combination on the ARCI (Drug: *F* = 0.243, *P* = 0.623, Drug × Subscale: *F* = 0.374, *P* = 0.827, Drug × Dose × Subscale: *F* = 0.560, *P* = 0.809, Figure 3) or the POMS (Drug: *F* = 0.976, *P* = 0.325, Drug × Subscale: *F* = 0.250, *P* = 0.959, Drug × Dose × Subscale: *F* = 0.0361, *P* = 1.000, Figure 4).

Side effects of oxycodone and the combination of oxycodone plus the antipsychotic were similar in nature and did not differ between oxycodone alone or when combined with the antipsychotics. The reported side effects were sedation (*n* = 8, 53.3%), nausea (*n* = 7, 46.7%), light-headedness (*n* = 7, 46.7%), vomiting (*n* = 4, 26.7%), hot/sweaty (*n* = 3, 20%), dry mouth (*n* = 2, 13.3%), racing heart (*n* = 1, 6.7%), and headache (*n* = 1, 6.7%).

## Discussion and Conclusions

It can be inferred from the data obtained in this preliminary study that the combination of an atypical antipsychotic with oxycodone did not alter the analgesic response of oxycodone or increase the incidence of adverse effects when compared to oxycodone alone. Such information is critical as it provides the foundation for future studies of abuse potential in recreational drug users. Although 75% (3 out 4) of the subjects experienced common adverse events, specifically nausea and vomiting in response to oxycodone (15 mg), this occurred in both placebo and drug combination groups. At lowered oxycodone dosages with fixed antipsychotic dosages, there was a dramatically reduced (eliminated) incidence of nausea and vomiting observed, with either oxycodone alone or the drug combinations. It is well-established that healthy subjects are more sensitive to the adverse effects of opioids than recreational drug users or chronic pain patients, due to the rapid tolerance of these effects (16). This likely accounts for the high incidence of nausea and vomiting seen in these subjects which is not seen frequently with a dose of 15 mg oxycodone in chronic pain patients. These data suggest that the risks of the off-label use of these drugs are not greater than those for the approved indications (i.e., pain for oxycodone and psychosis for risperidone and ziprasidone).

Given that opioids and antipsychotics are both central nervous system (CNS) depressants, we used the POMS to examine whether the combination exacerbated somnolence. There were no significant effects of the drug combination on the fatigue, confusion, or vigor subscales when compared to oxycodone alone.

To examine drug liking and disposition to take again, we administered the DEQ. Unfortunately, we were unable to observe dose-dependent increases in drug liking following oxycodone administration in this patient population. At the higher doses, the side effects such as nausea and vomiting may have been confounding factors that influenced the likability. Thus, it was not possible to examine whether the drug combination indeedy reduced the abuse liability of oxycodone. This was not entirely unanticipated as subjects with an opioid use disorder were explicitly excluded from the study. To better understand whether the drug combination reduces the abuse liability of oxycodone requires studies in drug users. Such a study is currently in progress (FDA IND:141,031 Clinicaltrials.gov Protocol # HSC20180167, NCT03837860).

As mentioned above, our study intentionally utilized two different antipsychotics—risperidone because of its metabolism and ziprasidone because of its side effect profile. This is a limitation of our study because of decreased ability to generalize the findings. Nevertheless, we believe that our conclusions listed above are appropriate for this pilot data.

Taken together, these data demonstrate that the addition of an atypical antipsychotic does not interfere with the analgesic effect of oxycodone, and that the risks of the off-label use of these drugs are not greater than those for the approved indications (i.e., pain for oxycodone and psychosis for risperidone and ziprasidone). These studies provide the foundation for future studies (currently underway) to examine the effect of the combination on abuse liability in recreational drug users. If effective, this approach could be beneficial in the management of opioid treatments for disorders such as chronic pain.

## Data Availability Statement

The original contributions presented in the study are included in the article/supplementary material, further inquiries can be directed to the corresponding author/s.

## Ethics Statement

The studies involving human participants were reviewed and approved by UTHSCSA Internal Review Board. The patients/participants provided their written informed consent to participate in this study.

## Author Contributions

AN, DL, JP, AF, RT, MC, AB, and ME contributed to the conception and the design of the study. RT and AB organized the database. DL performed statistical analysis. DL and AB wrote the first draft of the manuscript. All authors contributed to manuscript revision and read and approved the submitted version.

## Funding

This research was supported by grants provided by CBN/CTSA (Center for Biomedical Neuroscience/Clinical and Translational Science Award), and PTEF (The President's Translational and Entrepreneurial Research Fund) from UT Health SA.

## Conflict of Interest

DL and AF have a patent filed on this work. The remaining authors declare that the research was conducted in the absence of any commercial or financial relationships that could be construed as a potential conflict of interest.

## Publisher's Note

All claims expressed in this article are solely those of the authors and do not necessarily represent those of their affiliated organizations, or those of the publisher, the editors and the reviewers. Any product that may be evaluated in this article, or claim that may be made by its manufacturer, is not guaranteed or endorsed by the publisher.
